# miRNA-520c-3p accelerates progression of nasopharyngeal carcinoma via targeting RAB22A

**DOI:** 10.3892/ol.2020.12182

**Published:** 2020-10-01

**Authors:** Xiaohan Sun, Wenrui Xu, Chuanshan Zang, Na Li

Oncol Lett 19: 771-776, 2020; DOI: 10.3892/ol.2019.11144

Following the publication of this article, the authors have contacted the Editor to request its retraction on account of having been unable to reproduce the same results on performing a further series of experiments.

The Editor has agreed to the authors' request, and therefore this article has been retracted from *Oncology Letters*. All the authors agree to this retraction. The authors apologize to the readership of the Journal for any inconvenience caused.

